# Supplementary material for: Effect of Transgenesis on mRNA and miRNA Profiles in Cucumber Fruits Expressing Thaumatin II
Source: Genes (Basel). 2020 Mar 20;11(3):334. doi: 10.3390/genes11030334 (PMC7140888; doi:10.3390/genes11030334)
Supplement: Supplementary file 1 [file genes-11-00334-s001.zip › S8 Results of the STRING molecular network for NF-Y.pdf]

Supplementary materials - Results of the SRING molecular network for NF-Y transcription factor (NF-YA10) according to *A. thaliana* database. Blue is Histone-like TF domain (PF00808) and green is core histone domain (PF00125). Blue/green nodes present proteins with both domains (PF00808 and PF00125)

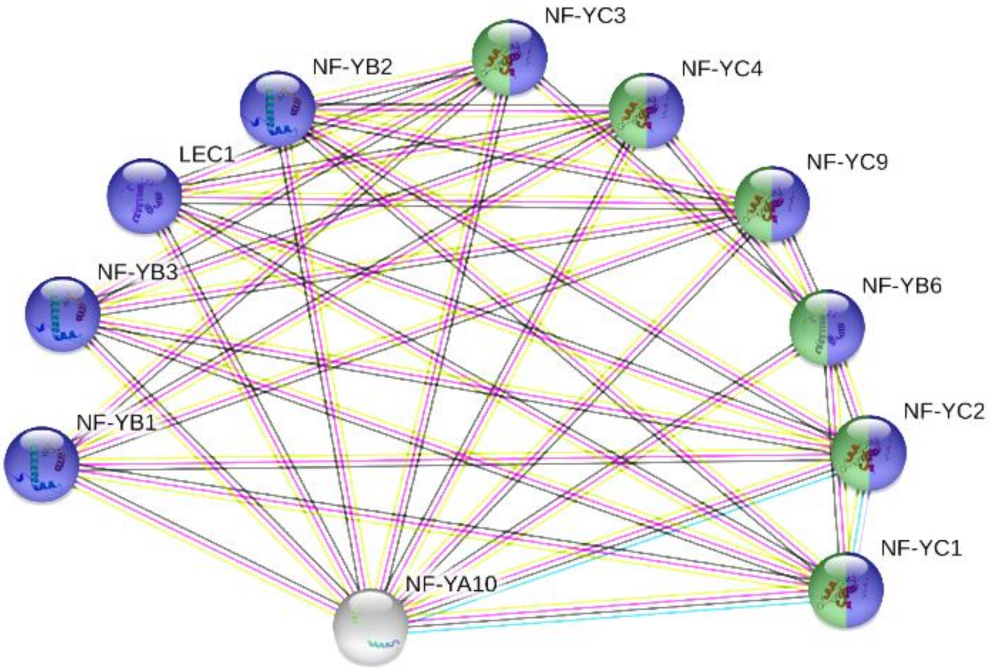

Network Stats

number of nodes:	11	expected number of edges:	10
number of edges:	36	PPI enrichment p-value:	2.54e-10
average node degree:	6.55		
avg. local clustering coefficient:	0.42		

your network has significantly more interactions than expected (what does that mean?)

Functional enrichments in your network

Note: some enrichments may be expected here (why?)

Biological Process (GO)			
GO-term	*description*	*count in gene set*	*false discovery rate*
GO:2000306	positive regulation of photomorphogenesis	4 of 6	4.18e-11
GO:0051252	regulation of RNA metabolic process	11 of 2209	4.18e-11
GO:0006355	regulation of transcription, DNA-templated	11 of 2167	4.18e-11
GO:0006351	transcription, DNA-templated	11 of 1957	4.18e-11
GO:0016070	RNA metabolic process	11 of 3007	1.66e-10
			(more ...)

Molecular Function (GO)			
GO-term	*description*	*count in gene set*	*false discovery rate*
GO:0046982	protein heterodimerization activity	10 of 103	1.54e-22
GO:0003677	DNA binding	11 of 1983	1.46e-12
GO:0003700	DNA-binding transcription factor activity	10 of 1333	3.03e-12
GO:0043565	sequence-specific DNA binding	6 of 730	2.26e-07
GO:0003712	transcription coregulator activity	2 of 70	0.00046

Cellular Component (GO)			
GO-term	*description*	*count in gene set*	*false discovery rate*
GO:0005634	nucleus	11 of 4229	2.39e-08
GO:0016602	CCAAT-binding factor complex	3 of 11	1.74e-07

Reference publications			
publication	*(year) title*	*count in gene set*	*false discovery rate*
PMID:21738795	(2011) Identification and characterization of NF-Y transcrip...	10 of 21	3.24e-27
PMID:25542200	(2015) Genome-wide expression analysis of soybean NF-Y ...	10 of 23	3.38e-27
PMID:22489162	(2012) Homologous NF-YC2 subunit from Arabidopsis and ...	10 of 29	1.55e-26
PMID:23527203	(2013) NUCLEAR FACTOR Y transcription factors have both...	10 of 31	2.05e-26
PMID:28119722	(2016) The Arabidopsis thaliana Nuclear Factor Y Transcrip...	10 of 53	1.87e-24
			(more ...)

UniProt Keywords			
keyword	*description*	*count in gene set*	*false discovery rate*
KW-0010	Activator	10 of 497	4.16e-16
KW-0238	DNA-binding	10 of 1902	1.23e-10
KW-0805	Transcription regulation	10 of 1996	1.32e-10
KW-0539	Nucleus	10 of 3725	3.77e-08
KW-0938	Abscisic acid signaling pathway	3 of 154	4.40e-05

PFAM Protein Domains			
domain	*description*	*count in gene set*	*false discovery rate*
PF00808	Histone-like transcription factor (CBF/NF-Y) and archaeal hi...	10 of 38	3.26e-27
PF00125	Core histone H2A/H2B/H3/H4	6 of 51	4.22e-14

INTERPRO Protein Domains and Features			
domain	*description*	*count in gene set*	*false discovery rate*
IPR003958	Transcription factor CBF/NF-Y/archaeal histone domain	10 of 27	2.89e-28
IPR009072	Histone-fold	10 of 75	1.30e-24
IPR003956	Transcription factor, NFYB/HAP3, conserved site	5 of 10	1.79e-14
