# Supplementary material for: Effect of Transgenesis on mRNA and miRNA Profiles in Cucumber Fruits Expressing Thaumatin II
Source: Genes (Basel). 2020 Mar 20;11(3):334. doi: 10.3390/genes11030334 (PMC7140888; doi:10.3390/genes11030334)
Supplement: Supplementary file 1 [file genes-11-00334-s001.zip › S9 Results of the STRING molecular network for Annexin.pdf]

Supplementary materials - Results of the SRING molecular network for Annexin (ANNATA3) according to *A. thaliana* database. Blue - present calcium binding domain and red present proteins involved in plant – pathogen interaction.

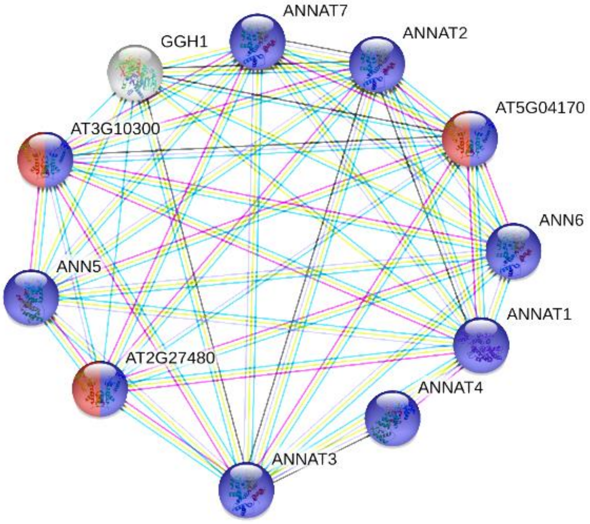

number of nodes:	11	expected number of edges:	10
number of edges:	47	PPI enrichment p-value:	1.11e-16
average node degree:	8.55		
avg. local clustering coefficient:	0.968		

your network has significantly more interactions than expected (what does that mean?)

Functional enrichments in your network

Note: some enrichments may be expected here (why?)

Biological Process (GO)

GO-term	description	count in gene set	false discovery rate
GO:0009408	response to heat	7 of 184	2.57e-11
GO:0009414	response to water deprivation	7 of 292	3.04e-10
GO:0009409	response to cold	7 of 347	4.98e-10
GO:0009651	response to salt stress	7 of 492	4.40e-09
GO:0009639	response to red or far red light	2 of 186	0.0184

(more ...)

Molecular Function (GO)

GO-term	description	count in gene set	false discovery rate
GO:0005544	calcium-dependent phospholipid binding	7 of 11	2.40e-19
GO:0005509	calcium ion binding	10 of 226	5.32e-19
GO:0004198	calcium-dependent cysteine-type endopeptidase activity	2 of 3	9.68e-06
GO:0070011	peptidase activity, acting on L-amino acid peptides	3 of 394	0.0020

Reference publications

publication	(year) title	count in gene set	false discovery rate
PMID:23133603	(2012) Genome-wide comparative analysis of annexin supe...	7 of 11	9.57e-19
PMID:23750279	(2013) A cotton annexin protein AnxGb6 regulates fiber elo...	7 of 23	3.06e-17
PMID:27236332	(2016) Comprehensive analyses of the annexin gene family...	6 of 14	2.13e-15
PMID:28935951	(2017) Overexpression of annexin gene AnnSp2, enhances ...	5 of 12	1.16e-12
PMID:28422051	(2017) Annexins as Overlooked Regulators of Membrane Tr...	5 of 40	1.83e-10

(more ...)

KEGG Pathways

pathway	description	count in gene set	false discovery rate
ath04626	Plant-pathogen interaction	3 of 165	7.19e-05

Reactome Pathways			
pathway	*description*	*count in gene set*	*false discovery rate*
ATH-109582	Hemostasis	9 of 154	8.47e-18
ATH-6798695	Neutrophil degranulation	10 of 533	9.68e-16
ATH-114608	Platelet degranulation	6 of 50	1.06e-13
ATH-1474228	Degradation of the extracellular matrix	3 of 9	2.77e-08
ATH-425561	Sodium/Calcium exchangers	3 of 10	2.88e-08
			(more ...)

UniProt Keywords			
keyword	*description*	*count in gene set*	*false discovery rate*
KW-0111	Calcium/phospholipid-binding	7 of 8	1.47e-20
KW-0041	Annexin	7 of 8	1.47e-20
KW-0106	Calcium	10 of 437	6.93e-17
KW-0677	Repeat	10 of 3072	1.25e-08
KW-0007	Acetylation	6 of 840	1.10e-06
			(more ...)

PFAM Protein Domains			
domain	*description*	*count in gene set*	*false discovery rate*
PF00191	Annexin	7 of 8	5.50e-21
PF13202	EF hand	2 of 119	0.0031

INTERPRO Protein Domains and Features			
domain	*description*	*count in gene set*	*false discovery rate*
IPR037104	Annexin superfamily	7 of 8	1.01e-20
IPR018502	Annexin repeat	7 of 8	1.01e-20
IPR001464	Annexin	7 of 8	1.01e-20
IPR018252	Annexin repeat, conserved site	6 of 7	3.69e-18
IPR009118	Annexin D, plant	4 of 5	3.88e-12
			(more ...)
